# Experimental and Statistical Evaluation of the Size Effect on the Bending Strength of Dimension Lumber of Northeast China Larch

**DOI:** 10.3390/ma9020089

**Published:** 2016-01-30

**Authors:** Yong Zhong, Hai-Qing Ren, Ze-Hui Jiang

**Affiliations:** 1 Research Institute of Wood Industry, Chinese Academy of Forestry, Beijing 100091, China; zhongy@caf.ac.cn (Y.Z.); renhq@caf.ac.cn (H.-Q.R.); 2 International Center for Bamboo and Rattan, Beijing 100102, China

**Keywords:** size effect, bending strength, dimension lumber, Northeast China larch (*Larix gmelinii*)

## Abstract

This study investigated the size effect on the bending strength (modulus of rupture—MOR) of dimension lumber of Northeast China larch (*Larix gmelinii*); providing a basis for the further application in light wood frame construction. Experimental and statistical evaluations were conducted on the bending strength. A total of 2409 full-size dimension lumber samples were tested by static bending tests; which included three different sizes: 2 × 3; 2 × 4; and 2 × 6. Results indicate that the size has a significant effect on the MOR. Both the chi-square (χ^2^) and Kolmogorov-Smirnov (K-S) test results show that the lognormal distribution generally fits to the MOR better than to the normal distribution. Additionally; the effects of partial safety factor (γ_R_) and live-to-dead load ratio (ρ) were studied by reliability analysis. Reliability analysis results indicate that the reliability index increases nonlinearly with the decrease of γ_R_ and the rise of ρ. Finally; the design value of bending strength and its adjusting factor of size effect of 2 × 3; 2 × 4; and 2 × 6 larch dimension lumber were obtained according to the Chinese National Standards’ requirements of the reliability index.

## 1. Introduction

Chinese larch (*Larix gmelinii*), an abundant wood resource, comprises 55% of the wooded areas and 75% of forest stocks in China’s frigid temperate zone, and can be used quite extensively to fabricate dimension lumber, glued lumber, and wood-based composites due to its favorable mechanical properties [1]. Due to the general lack of research regarding product fabrication processes and technologies, however, dimension lumber products manufactured from China’s own tree species remain limited.

The mechanical performance of dimension lumber is generally determined by one of two common methods: full-size testing, or small clear wood specimen testing. Considering the acute necessity of accurate test results and the structural safety requirements in practice, the full-size test method substituting the small clear wood specimens test method, has been adopted widely in most developed countries, such as the United States, Canada, and Japan [2,3]. However, the design value of structural wooden materials, fabricated from China’s native tree species, is still determined by the traditional small clear wood specimens test method.

Thus in an effort to ensure the safe use of dimension lumber and to promote the development of light wood frame construction, the mechanical performance of dimension lumber using China’s own Larch forestry resources was studied by the full-size test method during the 11th Five-Year Plan of China. The Chinese government and a handful of research institutions also began promoting the use of larch in light wood frame construction around this time. Several works [4,5,6,7] have shown that mechanical strength is reduced with the increase of the size and the decrease of strength grade. In addition, the effects of various testing methods on the mechanical properties of dimension lumber have been investigated [8] as well as the relationships between the visual defects, such as knots, slope of grain, checks, and splits, and the mechanical strength of larch dimension lumber [9,10,11]. Research has shown that analysis via the lognormal distribution can provide a good fit for strength properties [12]. Based on reliability analysis, the design value of the compression strength parallel to grain and of the bending strength for larch 2 × 4 lumber has been effectively determined [13,14]. Both the design value and its adjusting factor, as far as mechanical performance, are crucial considerations for the application of wood material in building construction; unfortunately, these have yet to be fully realized for China’s lumber resources. To this effect, it remains necessary to determine the adjusting factor of the mechanical properties and the related design value based on full-size test results.

Therefore, the objective of this study was to investigate the size effects on the bending strength (MOR) of dimension lumber of Northeast China larch (*Larix gmelinii*), providing a basis for its further application in light wood frame construction. An extensive experimental study was conducted. Besides, the statistical analyses of MOR were also performed.

## 2. Materials and Methods

### 2.1. Materials

Larch (*Larix gmelinii*) was collected from the Cuigang and Pangu forest farms of Heilongjiang Province, China. The diameter range of logs was 160–340 mm, and the average tree age was 35 years. The material was cut into 2 × 3, 2 × 4, and 2 × 6 dimension lumber samples. As specified in the Chinese National Standard [15], any single digit of lumber size, measured in millimeters (mm), was rounded to zero or five; unlike other countries’ standards, then, the actual size of the 2 × 3, 2 × 4, and 2 × 6 lumber specimens were 40 × 65, 40 × 90, and 40 × 140 mm, respectively. The length of each specimen was 4000 mm. The strength grade of dimension lumber is determined by its visual defects, such as knots, slope of grain, checks, splits and so on, according to the NLGA standard [16]. Besides, grades SS, No. 1, No. 2, and No. 3 in the NLGA standard, which are referred to in this paper, can be equated to grades Ic, IIc, IIIc, and IVc, respectively, in the Chinese National Code [15].

The number of specimens, the mean value, and coefficient of variation (COV) of density for each size of dimension lumber are shown in Table 1. Due to lack of a sufficient number of specimens to obtain accurate results, grades No. 1 and No. 3 of 2 × 6 lumber were not analyzed in this paper. According to ASTM D245 [17], an assumed minimum grade quality index (GQI) of each test sample was determined by the above visual defects.

Before bending testing, all specimens were conditioned at 20 °C and 65% relative humidity (RH) in a standard room, to arrive at the equilibrium moisture content (EMC). The measured average moisture content was 11.3% with a standard deviation of 1.11% [18].

### 2.2. Static Testing Method

According to ASTM D4761 [19], the third-point edgewise bending tests were carried out on an MTS universal testing machine. Specimens were loaded at a rate of 5 mm/min, which continued until failure. The span to depth ratio was 18 to 1. The MOR of each specimen was calculated as follows:

(1) $$\mathrm{MOR}=aF_{\max}/2W$$

where *a* is the distance between a loading position and the nearest support (mm), *F*_max_ is the maximum load (N), and *W* is the section modulus (mm^3^). 

The MOR of each specimen, adjusted to 15% MC (MOR_15_) in accordance with ASTM D1990 [20], can be represented as follows:

(2) $${\mathrm{MOR}}_{15}=\{\begin{array}{c} \mathrm{MOR}\\ \mathrm{MOR}+(M_{1}-15)\times \left(\mathrm{MOR}-16.66\right)/\left(40-M_{1}\right) \end{array}\begin{array}{c} \begin{array}{c} \mathrm{MOR}\leq 16.66\mathrm{MPa}\\ \mathrm{MOR}>16.66\mathrm{MPa} \end{array} \end{array}$$

where *M*_1_ is the moisture content of the specimen (%).

**Table 1:** Number of specimens and density of each dimension lumber size. COV**:** Coefficient of variation

Dimension	Grade	Number of Specimens	Density	
			Mean (kg/m^3^)	COV (%)
2 × 3	SS	209	649	10.48
	No. 1	109	632	10.55
	No. 2	255	635	11.29
	No. 3	281	647	11.30
2 × 4	SS	429	646	10.69
	No. 1	201	632	10.60
	No. 2	285	639	10.66
	No. 3	165	648	11.29
2 × 6	SS	253	611	8.58
	No. 1	28	611	9.89
	No. 2	134	631	60.86
	No. 3	60	653	11.11

### 2.3. Statistical Analysis

#### 2.3.1. One-Way Anova Analysis

The graphical analysis was conducted with Origin 9 software (OriginLab Corporation, Northampton, MA, USA). The differences in bending strength between different sizes were analyzed by one-way analysis of variance (ANOVA); multiple comparisons for different sizes were calculated by the Least Significant Difference (LSD) method using the ANOVA test results using SPSS 19.0 (IBM SPSS Corporation, Chicago, IL, USA). Significance level was set to 0.05.

#### 2.3.2. Distribution Model

Both chi-square (χ^2^) and Kolmogorov-Smirnov (K-S) tests were used to determine the probability distribution of MOR_15_.

For the chi-square (χ^2^) test method, the probability distribution of MOR_15_ was assumed to be either a normal distribution (*N*), or a lognormal distribution (*L*). The cumulative distribution function is defined as follows:

(3) $$F_{0}(x)=\frac{1}{\sqrt{2\pi }\sigma }\int_{-\infty }^{x}e^{-\frac{{\left(t-\mu \right)}^{2}}{2\sigma^{2}}}dt$$

where *x* and *t* are random variables of either MOR_15_ or logarithmic MOR_15_ obtained by the static bending test, and μ and σ are unknown parameters determined in the following analysis.

First, the μ and σ parameters were calculated by the maximum likelihood method in which the likelihood function can be expressed:

(4) $$L\left(\mu ,\sigma^{2}\right)=\prod_{i=1}^{n}\frac{1}{\sqrt{2\pi }\sigma }e^{-\frac{{\left(x_{i}-\mu \right)}^{2}}{2\sigma^{2}}}={\left(2\pi \sigma^{2}\right)}^{-\frac{n}{2}}e^{-\frac{1}{2\sigma^{2}}\sum_{i=1}^{n}{\left(x_{i}-\mu \right)}^{2}}$$

where *x_i_* is the random variable of the *i*th sample and *n* is the total number of samples.

After a logarithmic transformation, the likelihood function can be rewritten as follows:

(5) $$\log L\left(\mu ,\sigma^{2}\right)=-\frac{n}{2}\log\left(2\pi \right)-\frac{n}{2}\log\left(\sigma^{2}\right)-\frac{1}{2\sigma^{2}}\sum_{i=1}^{n}{\left(x_{i}-\mu \right)}^{2}$$

By calculating the partial derivatives of Equation (5), the likelihood equations can be obtained, which are defined as:

(6) $$\{\begin{array}{c} \frac{\partial \log L\left(\mu ,\sigma^{2}\right)}{\partial \mu }=\frac{1}{\sigma^{2}}\sum_{i=1}^{n}\left(x_{i}-\mu \right)=0\\ \frac{\partial \log L\left(\mu ,\sigma^{2}\right)}{\partial \sigma^{2}}=-\frac{n}{2\sigma^{2}}+\frac{1}{2\sigma^{4}}\sum_{i=1}^{n}{\left(x_{i}-\mu \right)}^{2}=0 \end{array}$$

The maximum likelihood estimation of the μ and σ parameters is as follows:

(7) $$\{\begin{array}{c} \hat{\mu }=\bar{x}=\frac{1}{n}\sum_{i=1}^{n}x_{i}\approx \frac{1}{N}\sum_{j=1}^{k}n_{i}\bar{x_{i}}\\ \hat{\sigma }=\sqrt{\frac{1}{n}{\sum_{i=1}^{n}\left(x_{i}-\bar{x}\right)}^{2}}\approx \sqrt{\frac{1}{N}\sum_{j=1}^{k}n_{i}{\left(\bar{x_{i}}-\bar{x}\right)}^{2}} \end{array}$$

where *k* is the sum of interval numbers of observed MOR_15_, *N* is the total number of specimens equal to the sum of *n_i_*, *n_i_* is the observed number of specimens located in the *i*th interval, and $\bar{x_{i}}$ is the value of the interval center.

By substituting Equation (7) into Equation (3), *F*_0_(*x*) can be rewritten as:

(8) $$F_{0}(x)=\frac{1}{\sqrt{2\pi }\hat{\sigma }}\int_{-\infty }^{x}e^{-\frac{{\left(t-\hat{\mu }\right)}^{2}}{2{\hat{\sigma }}^{2}}}dt$$

*F*(*x*) is the cumulative distribution function of MOR_15_ obtained by the static bending test. Whether or not the *F*(*x*) is suitable for the assumed *F*_0_(*x*) at the setting level, can be judged by the chi-square (χ^2^) test [21]. The calculation formula for χ^2^ is as follows:

(9) $$\chi^{2}=\sum_{i=1}^{k}\frac{{\left(n_{i}-N{\hat{p}}_{i}\right)}^{2}}{N{\hat{p}}_{i}}$$

where ${\hat{p}}_{i}$ and $N{\hat{p}}_{i}$ are the cumulative distribution probability and predicted numbers of random variables on the *i*th interval, respectively.

The formula for the Kolmogorov-Smirnov (K-S) test method is as follows:

(10) $$D=\left|S_{n}\left(x\right)-F_{0}\left(x\right)\right|$$

where *D* is the maximum absolute difference between the empirical distribution function and the theoretical distribution function, and $S_{n}\left(x\right)$ is the empirical distribution function of *x*.

#### 2.3.3. Reliability Analysis

As reported in the authors’ previous research [13,14], the characteristic values of MOR_15_ for larch lumber can be determined by the fifth percentile value of probability distribution. As a lognormal distribution, the characteristic value (*f*_k_) and design value (*f*_d_) are as follows [22,23]:

(11) $$f_{k}=e^{\mu_{f}\left(1-1.645\delta_{f}\right)}$$

(12) $$f_{d}=f_{k}/\gamma_{f}=\mu_{k3}f_{k}/\gamma_{R}$$

where μ_f_ is the mean value of logarithmic MOR_15_, and δ_f_ is the coefficient of variance (COV) (Table 2). γ_f_ is the partial safety factor of material property, µ_k3_ is the mean value of the adjusting factor for the effect of long-term load and is equal to 0.72, and γ_R_ is the partial safety factor of bending resistance.

**Table 2:** Summary of bending strength statistics adjusted to 15% moisture content (MOR_15_).

Grade	Size	MOR_15_			ln(MOR_15_)		
		Mean (MPa)	SD (MPa)	COV (%)	Mean (MPa)	SD (MPa)	COV (%)
SS	2 × 3	72.1	23.1	32.1	4.22	0.352	8.34
	2 × 4	65.1	20.3	31.2	4.13	0.330	7.99
	2 × 6	55.6	19.5	35.0	3.95	0.370	9.36
No. 1	2 × 3	56.3	20.9	37.1	3.96	0.385	9.71
	2 × 4	46.9	16.5	35.1	3.79	0.356	9.41
No. 2	2 × 3	56.6	20.2	35.7	3.97	0.379	9.54
	2 × 4	51.9	18.9	36.4	3.88	0.376	9.70
	2 × 6	49.9	20.4	40.9	3.83	0.409	10.7
No. 3	2 × 3	51.6	21.4	41.4	3.85	0.439	11.4
	2 × 4	49.7	20.9	42.1	3.81	0.449	11.8

In order to determine the design value of the bending strength and its adjusting factor of size effect of larch lumber, first-order second-moment reliability analyses were performed for all data cells and simulation load cases: dead load plus office occupancy load (*G* + *L*_O_), dead load plus residential occupancy load (*G* + *L*_R_), dead load plus wind load (*G* + *L*_W_), and dead load plus snow load (*G* + *L*_S_) [22,24]. A calculation program for reliability index (β) was developed in Matlab 7 software (MathWorks Corporation, Natick, MA, USA). 

The performance function can be expressed as follows [25,26,27]:

(13) Z=R−(D+L)

where *R*, *D*, and *L* are random variables representing bending resistance, dead load (*G*), and live load (*L*_O_, *L*_R_, *L*_W_, or *L*_S_), respectively.

## 3. Results and Discussion 

### 3.1. Results of the Bending Test

Each test sample met grade quality index (GQI) requirements, under which the difference between the observed GQI of the samples and the assumed GQI of the grade must be 5% or less of the total range of possible GQI [20]. An observed GQI for each test sample can be calculated for all specimens that did not fail in clear wood [17,20]. The difference between the observed GQI of the samples and the assumed GQI of the grade was less than 5%. Thus, the observed GQI of test data could be used to represent the grades of the samples.

Table 2 shows the mean value, standard deviation, and coefficient of variation (COV) of the final MOR_15_ for each size of lumber. Results indicated that the MOR_15_ is correlated to the size. The MOR_15_ values showed highly significant (*p* < 0.05, *F*-test ANOVA) differences between different sizes of dimension lumber, except pairs 2 × 3 *vs.* 2 × 4 of grade No. 3, and 2 × 4 *vs.* 2 × 6 of grade No. 3 (Table 3). For the same grade of lumber, the bigger the size, the smaller the MOR_15_. For instance, the mean value of MOR_15_ of grade SS for 2 × 3 was 72.1 MPa, 1.11, 1.30 times that of 2 × 4, and 2 × 6, respectively. Similar results were obtained in previous studies [4,28].

**Table 3:** One-way analysis of variance (ANOVA) test results of MOR_15_ for different lumber sizes.

Grade	Significance		
	2 × 3 *vs.* 2 × 4	2 × 3 *vs.* 2 × 6	2 × 4 *vs.* 2 × 6
SS	**	***	***
No. 1	***	-	-
No. 2	**	**	0.320
No. 3	0.300	-	-

The symbols ** and *** represent *p*-values less than 0.01 and 0.001, respectively.

### 3.2. Probability Distribution

**Figure 1:**
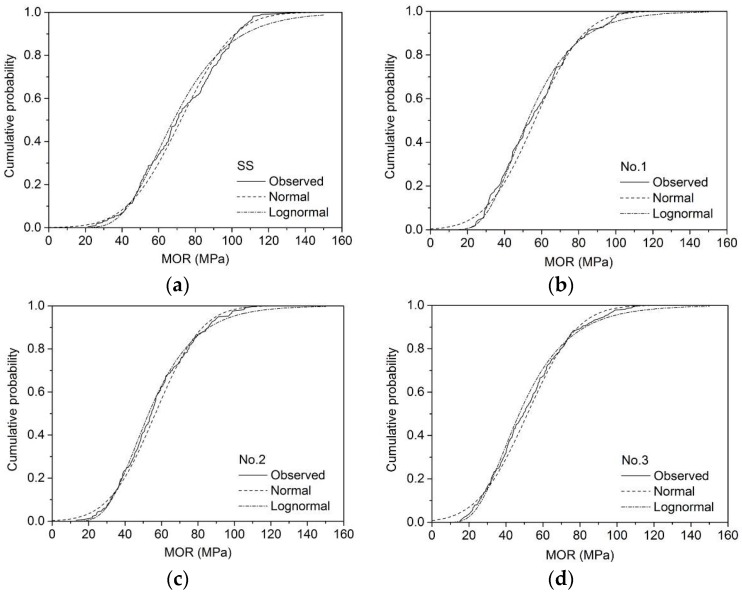
Normal and lognormal fit of MOR_15_ for 2 × 3 lumber: (**a**) SS; (**b**) No. 1; (**c**) No. 2; (**d**) No. 3.

**Figure 2:**
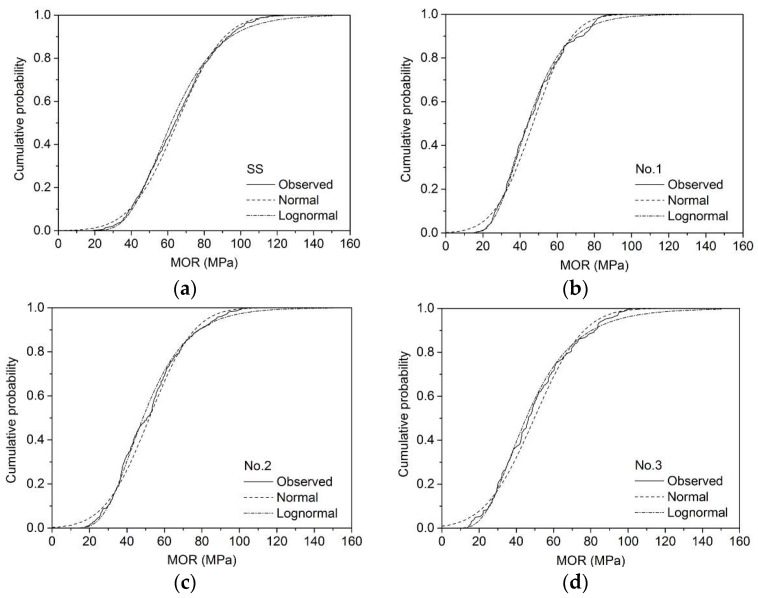
Normal and lognormal fit of MOR_15_ for 2 × 4 lumber: (**a**) SS; (**b**) No. 1; (**c**) No. 2; (**d**) No. 3.

**Figure 3:**
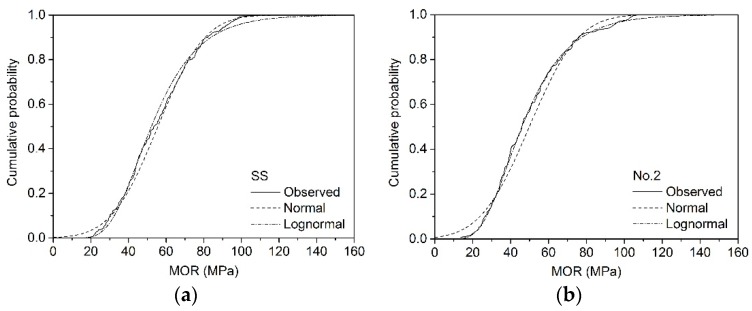
Normal and lognormal fit of MOR_15_ for 2 × 6 lumber: (**a**) SS; (**b**) No. 2.

The cumulative probability distributions of MOR_15_ for different sizes are presented in Figure 1, Figure 2 and Figure 3. The unknown parameters μ and σ were calculated according to Equations (4)–(8). For example, the estimated μ and σ of MOR_15_ of SS grade for 2 × 4 larch lumber were 65.1 and 20.3 MPa, while logarithmic MOR_15_ values were 4.12 and 0.328 MPa, respectively (Table 4).

**Table 4:** Calculation table of $\hat{\mu }$, $\hat{\sigma }$ and chi-square χ^2^ of MOR_15_ of SS grade larch 2 × 4 lumber.

Interval	Interval Center $\bar{x_{i}}$	Observed Numbers (*n_i_*)	Probability (${\hat{p}}_{i}$)		Predicted Numbers ($n{\hat{p}}_{i}$)	
			Normal	Lognormal	Normal	Lognormal
[15, 20]	17.5	1	0.0180	0.0003	7.7215	0.1228
[20, 25]	22.5	2	0.0235	0.0026	10.0771	1.1236
[25, 30]	27.5	5	0.0298	0.0109	12.7685	4.6816
[30, 35]	32.5	11	0.0366	0.0277	15.7082	11.9015
[35, 40]	37.5	25	0.0437	0.0508	18.7624	21.8053
[40, 45]	42.5	31	0.0507	0.0743	21.7583	31.8568
[45, 50]	47.5	38	0.0571	0.0923	24.4986	39.5891
[50, 55]	52.5	37	0.0624	0.1018	26.7815	43.6884
[55, 60]	57.5	35	0.0663	0.1028	28.4253	44.1028
[60, 65]	62.5	36	0.0683	0.0970	29.2922	41.5938
[65, 70]	67.5	41	0.0683	0.0867	29.3073	37.2154
[70, 75]	72.5	38	0.0664	0.0745	28.4693	31.9526
[75, 80]	77.5	32	0.0626	0.0619	26.8507	26.5543
[80, 85]	82.5	22	0.0573	0.0501	24.5873	21.5030
[85, 90]	87.5	23	0.0510	0.0398	21.8597	17.0551
[90, 95]	92.5	13	0.0440	0.0310	18.8692	13.3038
[95, 100]	97.5	14	0.0369	0.0239	15.8139	10.2396
[100, 105]	102.5	10	0.0300	0.0182	12.8678	7.7969
[105, 110]	107.5	8	0.0237	0.0137	10.1659	5.8860
[110, 115]	112.5	3	0.0182	0.0103	7.7976	4.4130
[115, 120]	117.5	2	0.0135	0.0077	5.8071	3.2907
[120, 125]	122.5	2	0.0098	0.0057	4.1988	2.4434
Sum	-	*N* = 429	-	-	402.388	422.120

Both the χ^2^ (Equation (9)) and K-S (Equation (10)) test values of different sizes of larch lumber are shown in Table 5. Results indicated that the lognormal distribution fitted the bending test data much better than the normal distribution for all sizes except the SS grade of 2 × 3, SS grade of 2 × 4, and No. 2 grade of 2 × 4. Findings presented by Dahlen *et al.* [29] similarly showed that the lognormal distribution model provides a better fit for Douglas fir and Southern pine 2 × 4 lumber.

The assumed *F*_0_(*x*) can be considered a good fit for the *F*(*x*) obtained by the static bending test when the critical value, which is dependent on probability levels, is greater than the test value [21]. At a probability level of 0.05, the χ^2^ test results showed that normal distribution fitting did not provide accurate results for most sizes, the exceptions being grade No. 1 of 2 × 3 and No. 3 of 2 × 4 larch lumber. Conversely, lognormal distribution fit the data well for grade SS of 2 × 4 (19.8 < 30.1), No. 1 of 2 × 3 (14.3 < 26.3) and 2 × 4 (17.6 < 22.4), No. 2 of 2 × 6 (11.3 < 27.6), and No. 3 of 2 × 3 (22.1 < 32.7) and 2 × 4 (14.8 < 26.3). When the probability level increased to 0.01, the lognormal distribution provided good fitting results for all sizes apart from SS of 2 × 3 and No. 2 of 2 × 4 (Table 5). The K-S test results showed that both the normal and lognormal distribution can provide a good fit at probability levels of 0.05 and 0.01. In this paper, lognormal distribution was thus selected for the following reliability analysis.

**Table 5:** χ^2^-values for bending strength adjusted to 15% moisture content (MOR_15_) for Chinese larch.

Grade	Size	χ^2^		$\chi_{a}^{2}$ = 0.05	$\chi_{a}^{2}$ = 0.01	*D*_max_		*D*_a_ = 0.05	*D*_a_ = 0.01
		Normal	Lognormal			Normal	Lognormal		
SS	2 × 3	33.08	45.29	32.67	38.93	0.066	0.087	0.094	0.113
	2 × 4	57.77	19.79	30.14	36.19	0.045	0.048	0.066	0.079
	2 × 6	29.67	29.15	27.59	33.41	0.068	0.057	0.086	0.102
No. 1	2 × 3	19.11	14.28	26.30	32.00	0.073	0.067	0.130	0.156
	2 × 4	33.51	17.62	22.36	27.69	0.078	0.037	0.096	0.115
No. 2	2 × 3	35.53	30.57	28.87	34.81	0.074	0.064	0.085	0.102
	2 × 4	39.45	89.28	25.00	30.58	0.075	0.070	0.081	0.097
	2 × 6	34.32	11.30	27.59	33.41	0.091	0.033	0.117	0.141
No. 3	2 × 3	35.40	22.05	32.67	38.93	0.066	0.054	0.081	0.097
	2 × 4	23.98	14.84	26.30	32.00	0.073	0.058	0.117	0.141

### 3.3. Reliability Analysis

Table 6 shows the characteristic values of MOR_15_ (*f*_k_) calculated by Equation (11). The *f*_k_ value generally decreased alongside the reduction in strength of size. For instance, the *f*_k_ for grade SS of 2 × 3 was 38.2 MPa, 0.061, 0.345 times higher than those of 2 × 4 and 2 × 6, respectively. The γ_R_ values were determined according to the reliability analysis described below.

**Table 6:** Characteristic values of MOR_15_.

Grade	SS			No. 1		No. 2			No. 3	
Size	2 × 3	2 × 4	2 × 6	2 × 3	2 × 4	2 × 3	2 × 4	2 × 6	2 × 3	2 × 4
*f*_k_ (MPa)	38.2	36.0	28.4	27.8	24.5	28.4	26.1	23.5	22.9	21.6

According to the authors’ previous research [13,14], random variables of bending resistance (*R*) are also distributed lognormally. The calculated mean value and COV of *R* for larch lumber are shown in Table 7 based on statistical theory.

**Table 7:** Summary of the resistance stress (*R*) statistics.

Grade	Size	*R* (MPa)		
		Mean Value	SD	COV (%)
SS	2 × 3	48.8	17.3	35.5
	2 × 4	44.1	15.3	34.8
	2 × 6	37.7	14.4	38.2
No. 1	2 × 3	38.1	15.3	40.1
	2 × 4	31.7	12.2	38.3
No. 2	2 × 3	38.3	14.9	38.8
	2 × 4	35.1	13.9	39.4
	2 × 6	33.8	14.7	43.6
No. 3	2 × 3	35.0	15.4	44.1
	2 × 4	33.6	15.0	44.8

**Figure 4:**
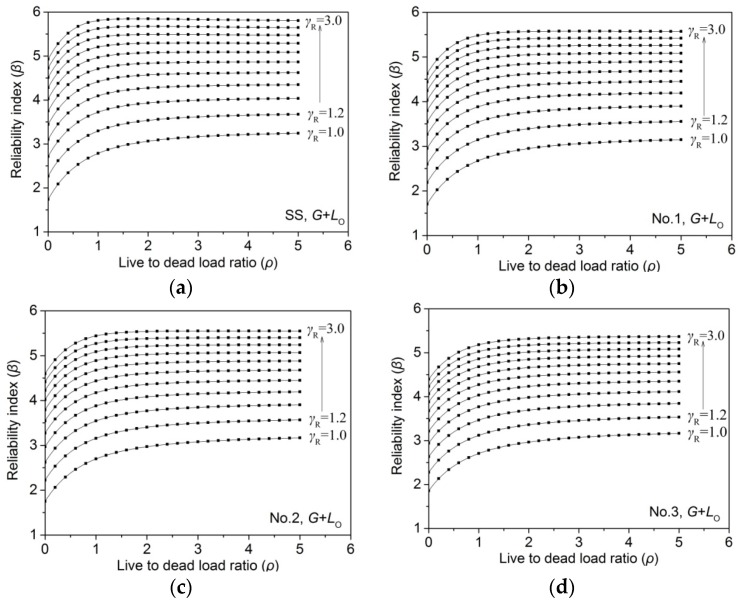
Relationship between reliability index (β) and live-to-dead load ratio (ρ) for larch 2 × 4 lumber under *G* + *L*_O_: (**a**) SS; (**b**) No. 1; (**c**) No. 2; (**d**) No. 3.

Reliability analysis results indicated that β increased nonlinearly as live-to-dead load ratio (ρ) increased in all the simulation load cases. As an example, the relationship between β and ρ of 2 × 4 larch lumber, under dead load (*G*) plus live office load (*L*_O_), are shown in Figure 4. Several previous studies [13,25,26,27,30] achieved a similar result regarding the relationship between ρ and β.

**Figure 5:**
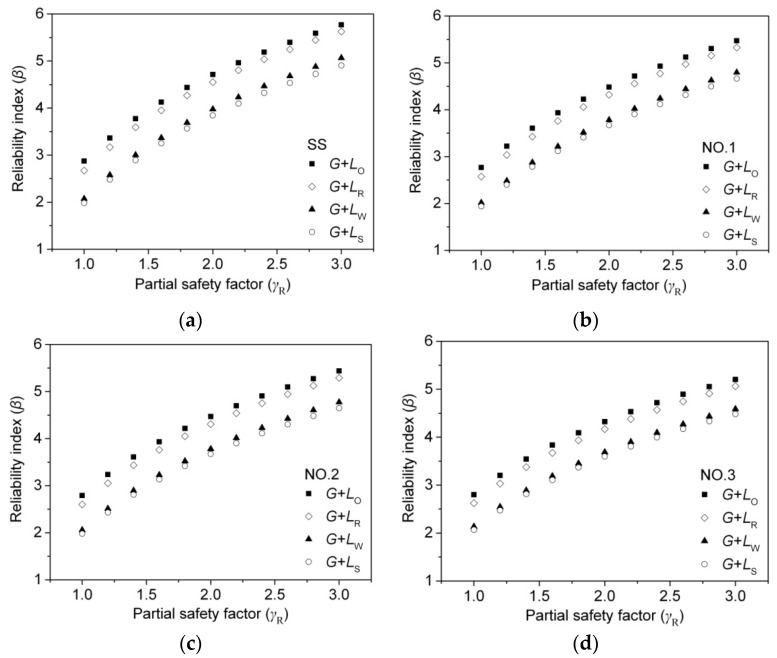
Relationship between reliability index (β) and partial safety factor (γ_R_) for larch 2 × 4 lumber under *G* + *L*_O_: (**a**) SS; (**b**) No. 1; (**c**) No. 2; (**d**) No. 3.

The reliability level must meet the target level (β_0_ = 3.2) to effectively determine the MOR_15_ design value [24]. In order to evaluate the reliability level, the live-to-dead load ratio (ρ) was specified as 0.25, 0.5, 0.75, 1.0, 1.25, 1.5, 1.75, 2.0, 2.25, 2.5, 2.75, and 3.0 for each load combination (*G* + *L*_O_, *G* + *L*_R_, *G* + *L*_W_, and *G* + *L*_S_), respectively, according to the Chinese National Standard [22], then the average of reliability index was calculated under different load ratios. The relationship between the average reliability index and the partial safety factor is shown in Figure 5.

**Figure 6:**
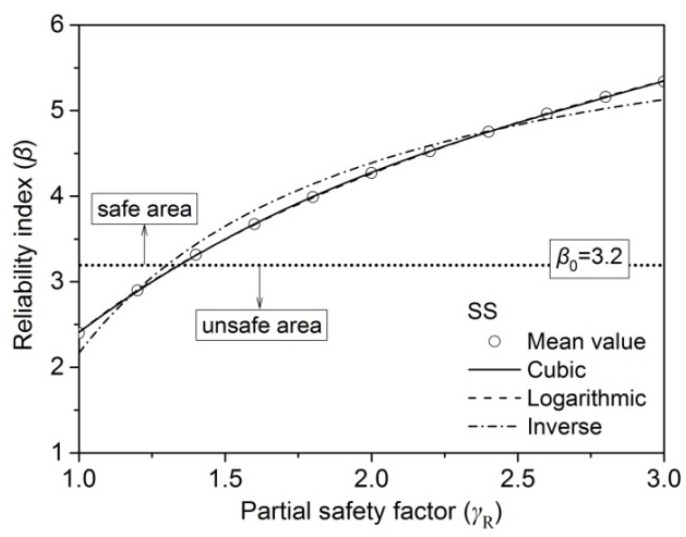
Prediction of reliability index (β) by partial safety factor (γ_R_) of grade SS for 2 × 4 lumber.

As shown in Figure 5, β increased nonlinearly with the increase of the partial safety factor (γ_R_) in all simulation load cases, *G* + *L*_O_, *G* + *L*_R_, *G* + *L*_W_, and *G* + *L*_S_. For the same ratio of live-to-dead load (ρ), the simulated load cases of the maximum and minimum β were *G* + *L*_O_ and *G* + *L*_S_, respectively. This is consistent with the findings of previous researchers [13,25,26,27]. The partial safety factor (γ_R_) of all sizes of larch lumber was obtained, as mentioned above, by taking the average of all simulation load cases. In order to obtain β, the linear, cubic, logarithmic, and inverse models were utilized to predict the relationship between β and γ_R_. For example, the predicted result of grade SS of 2 × 4 larch lumber is shown in Figure 6.

Fitting results indicated that the linear, cubic, logarithmic, and inverse models precisely predicted β as a function of γ_R_ for all sizes of larch lumber, though the logarithmic model (which was thus used for the reliability index calculation) fitted the data slightly better than other models. Table 8 shows all the fitting results for 2 × 4 larch lumber as an example, with standard errors of 0.006, 0.004, 0.004, and 0.002 for grades SS, No. 1, No. 2, and No. 3, respectively.

**Table 8:** Fitting results by various models for larch 2 × 4 lumber.

Grade	Model	Calculation Formula	*r*^2^	Std. Error	γ_R_ (β = β_0_ = 3.2)	*f*_d_ (MPa)
SS	Linear	*y* = 1.263 + 1.427*x*	0.975	0.159	1.357	19.1
	Logarithmic	*y* = 2.411 + 2.674ln*x*	1.000	0.006	1.342	19.3
	Inverse	*y* = 6.612 − (4.444/*x*)	0.978	0.150	1.303	19.9
	Cubic	*y* = −1.138 + 4.678*x* − 1.278*x*^2^ + 0.146*x*^3^	1.000	0.007	1.345	19.2
No. 1	Linear	*y* = 1.261 + 1.330*x*	0.976	0.147	1.458	12.1
	Logarithmic	*y* = 2.332 + 2.492*lnx*	1.000	0.004	1.417	12.5
	Inverse	*y* = 6.247 − (4.141/*x*)	0.977	0.142	1.360	13.0
	Cubic	*y =* −0.949 + 4.321*x* − 1.174*x*^2^ *+* 0.134*x*^3^	1.000	0.007	1.417	12.5
No. 2	Linear	*y* = 1.317 + 1.301*x*	0.976	0.143	1.447	13.0
	Logarithmic	*y* = 2.365 + 2.438*lnx*	1.000	0.004	1.408	13.4
	Inverse	*y* = 6.193 − (4.050/*x*)	0.977	0.139	1.354	13.9
	Cubic	*y* = −0.838 + 4.216*x* − 1.144*x*^2^ + 0.131*x*^3^	1.000	0.007	1.411	13.3
No. 3	Linear	*y* = 1.461 + 1.179*x*	0.976	0.129	1.475	10.5
	Logarithmic	*y* = 2.410 + 2.209*lnx*	1.000	0.002	1.429	10.9
	Inverse	*y* = 5.879 − (3.669/*x*)	0.977	0.127	1.369	11.4
	Cubic	*y* = −0.470 + 3.790*x* − 1.023*x*^2^ + 0.117*x*^3^	1.000	0.006	1.432	10.9

According to the above reliability analysis, plus the requirements for the minimum reliability index (β > β_0_ = 3.2) [22], the suggested design values of MOR_15_ and partial safety factors are shown in Table 9. For instance, the partial safety factors which satisfied the conditions γ*_R_* ≥ 1.34, 1.42, 1.41, and 1.43 for grades SS, No. 1, No. 2, and No. 3 of 2 × 4 larch lumber, respectively, can be considered safe for engineering design; the design values should be set to 19.3, 12.5, 13.4, and 10.9 MPa, as calculated by Equation (12). It is not reasonable, however, that *f*_d_ of grade No. 2 was larger than that of grade No. 1 in 2 × 3 and 2 × 4. To ensure conservative estimates and safe design, the *f*_d_ for grade No. 1 and No. 2 must have the small design value between them. Thus, the final design values of grade No. 2 in 2 × 3 and 2 × 4, which were 14.2, 13.4 MPa, respectively, were considered the same as that of grade No. 1.

Table 9 indicates that the size of larch lumber had significant impact on the design value of MOR_15_. For each grade of lumber, the bigger the size, the smaller the design value of MOR_15_. As an example, the design value of grade SS of 2 × 3 was 20.8 MPa, which was 1.08 and 1.40 times that of 2 × 4 and 2 × 6, respectively. It is important to note that, for convenience in practical engineering application, the design value specified in the national standard requires multiplying different adjusting factors by the same reference value for different sizes of the same grade of lumber. The design value of 2 × 3 larch lumber was selected as the reference value. 

**Table 9:** Design values of MOR_15_ and partial safety factor γ_R_.

Grade	SS			No. 1		No. 2			No. 3	
Size	2 × 3	2 × 4	2 × 6	2 × 3	2 × 4	2 × 3	2 × 4	2 × 6	2 × 3	2 × 4
*f*_d_ (MPa)	20.8	19.3	14.9	14.2	12.5	14.9	13.4	11.3	11.5	10.9
γ_R_	1.318	1.342	1.375	1.411	1.417	1.375	1.408	1.492	1.429	1.429

**Figure 7:**
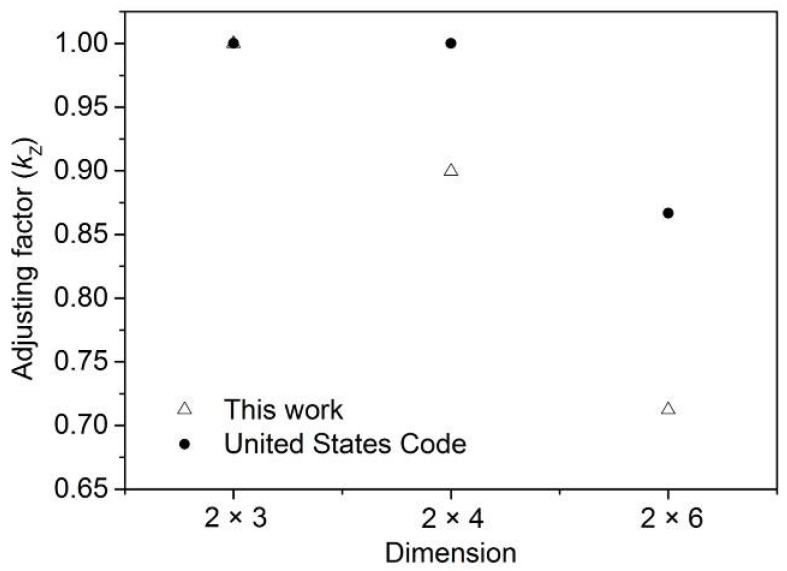
Adjusting factor of size effect (*k*_Z_) for larch lumber.

The adjusting factors of 2 × 4 were 0.925, 0.900, 0.900, and 0.942 for grades SS, No. 1, No. 2 and No. 3, and those of 2 × 6 were 0.713 and 0.762 for grades SS and No. 2, respectively. These results indicate that the adjusting factors were not the same among different grades, though most countries do set the same adjusting factors for different grades in one dimension. To ensure conservative estimates and safe design, it was reasonable to set adjusting factors to 0.900, 0.713 for 2 × 4 and 2 × 6 lumber, as shown in Figure 7. In the United States Code [2], adjusting factors are equal to 1.0 if the section height is not more than 90 mm, and grade and adjusting factors are uncorrelated. The adjusting factors of 2 × 4 and 2 × 6 are 1.00 and 0.867 for any tree species—significantly higher than those for larch lumber.

## 4. Conclusions

In this work, a series of static bending tests and statistical analyses were performed for Northeast China larch (*Larix gmelinii*) dimension lumber in order to investigate the size effect on the bending strength of dimension lumber. Frequency histograms of bending strength were fitted by normal and lognormal distribution models. Additionally, the effects of partial safety factor and live-to-dead load ratio were studied by reliability analysis. Based on the analysis of the test data, the following conclusions can be drawn:

- MOR_15_ is affected by the size of larch dimension lumber; the bigger the size, the smaller the MOR_15_.
- Design value of bending strength and adjusting factor of size effect of 2 × 3, 2 × 4, and 2 × 6 larch dimension lumber were successfully obtained according to the Chinese National Standards’ requirements.
- Further developments will be mainly devoted to carry out wider experimental activity particularly on more varied sizes and species, in order to validate the adjusting factor of size effect obtained in this work.
